# Domestic Violence Against Men in Canada: An Overlooked but Growing Public Health and Social Concern

**DOI:** 10.1177/15598276261448820

**Published:** 2026-05-05

**Authors:** Rudra Dahal, Aayush Dahal, Badri Karki

**Affiliations:** 1 Department of Community Health Sciences, Cumming School of Medicine, 70401 University of Calgary, Calgary, AB, Canada (RD); 2 Faculty of Science, 2129 University of Calgary, Calgary, AB, Canada (AD); 3 Faculty of Social Works, 2129 University of Calgary, Calgary, AB, Canada (BK)

**Keywords:** complications, mortality, quality

## Abstract

Domestic and intimate partner violence (IPV) in Canada is a complex public health and social issue affecting individuals of all genders, yet male victims remain significantly under-recognized. Although women experience the highest rates and most severe outcomes, growing evidence shows that men also face substantial physical, psychological, emotional, financial, and coercive abuse. Police-reported data from 2022 indicate that one in five IPV victims are male, with a 21% increase in IPV against men and boys since 2014. Social stigma, gender norms, and limited male-focused services contribute to under-reporting and inadequate support. Male victims often experience serious consequences, including mental health challenges, financial instability, strained relationships, and loss of child access. Addressing these gaps requires gender-inclusive services, awareness initiatives, and professional training to recognize and support male survivors. A comprehensive response that acknowledges all victims is essential for improving safety, reducing stigma, and strengthening Canada’s overall IPV prevention and intervention efforts.

“Public awareness campaigns are also critical to reduce stigma and improve recognition of male victimization.”

Domestic and intimate partner violence (IPV) in Canada is a complex social and public health issue affecting individuals across all genders. Although public discourse primarily focuses on women’s experiences, emerging evidence shows that men also face significant domestic violence that remains largely overlooked. Domestic violence is defined as a pattern of physical, sexual, psychological, emotional, or financial harm perpetrated by a partner or family member to exert control and dominance.^1^ While women experience the highest rates and most severe consequences of IPV, police-reported data from 2022 recorded more than 117,000 victims aged 12 and older, with approximately 1 in 5 being men.^2^ Longitudinal trends further indicate a 21% increase in IPV against men and boys from 2014 to 2022.^3^

Men experience a wide range of abusive behaviors, including physical violence such as hitting, slapping, or being attacked with objects.^4^ Psychological and emotional abuse, coercive control, financial manipulation, and sexual violence are also common.^4^ Coercive control—characterized by isolation, surveillance, intimidation, and domination—can be as harmful as physical assault. Financial abuse, including restricting access to money, sabotaging employment, or manipulating shared finances, has profound social and economic consequences.^4^ Men may additionally face legal or administrative abuse, such as false accusations or threats related to child custody or immigration status.^5^ These varied forms of abuse demonstrate that IPV against men extends beyond physical harm and encompasses multiple layers of control that affect mental, emotional, and social wellbeing.

Despite these realities, male victims remain significantly under-recognized. Societal expectations of masculinity—emphasizing strength, stoicism, and self-reliance—discourage men from seeking help.^5^ Many fear ridicule, disbelief, or being misidentified as the aggressor, particularly when their female partner appears distressed.^6^ These pressures contribute to substantial under-reporting and reinforce the invisibility of male victimization in research, policy, and service provision. Canada has extensive support systems for women, including shelters, counseling, and crisis lines, but men have limited access to safe housing, gender-sensitive counseling, or legal advocacy tailored to their needs.^5^ This service gap leaves many men trapped in abusive situations and perpetuates misconceptions that male IPV is rare or insignificant.

The consequences of IPV for men are severe. Psychological effects—including anxiety, depression, post-traumatic stress, substance use, and social withdrawal—are commonly reported.^7^ Many also experience financial instability, strained relationships, and challenges related to child custody.^7^ Long-term health consequences include chronic pain, injury-related complications, sleep disturbances, and reduced quality of life.^8^ Qualitative evidence further highlights social isolation, stigma, and difficulties maintaining relationships among male survivors.^9^ These impacts underscore that IPV against men is a significant public health issue with far-reaching personal and societal implications.

Recognizing male victims does not diminish the disproportionate harm faced by women; rather, it highlights the need for a more inclusive understanding of IPV. Violence is not exclusively gendered, and failing to acknowledge male experiences perpetuates stigma, reduces reporting, and limits the effectiveness of prevention and support programs.^9^ Canadian data reinforce this need: provinces such as Saskatchewan and Manitoba have seen notable increases in IPV involving men and boys.^3^ Surveys show that men experience both physical and psychological harm, including coercive control and financial abuse, and that many cases involve severe outcomes requiring policy attention.^2^

Addressing domestic violence against men requires evidence-based and gender-inclusive interventions. Training health care providers is essential, as limited knowledge and confidence can hinder effective identification and response.^10^ Integrating routine screening within health care settings may facilitate early detection and linkage to care.^11^ Expanding confidential and online support services can improve accessibility, particularly for individuals reluctant to disclose abuse.^12^ Tailored interventions that consider diverse backgrounds and circumstances have been shown to be more effective. Public awareness campaigns are also critical to reduce stigma and improve recognition of male victimization.^13^ Strengthening coordination across health care and social services is necessary to ensure comprehensive and timely support.^10^

In conclusion, domestic violence against men in Canada represents a significant yet often invisible public health and social issue. Despite rising prevalence, male victims face systemic barriers rooted in societal norms, stigma, and service gaps. Men experience a broad spectrum of abuse—physical, psychological, emotional, sexual, financial, and coercive control—each with serious personal and societal consequences. Effective solutions require expanding services, increasing awareness, promoting research, and challenging cultural assumptions about masculinity and victimhood. Only by acknowledging and responding to the experiences of all survivors can Canada build a more equitable and comprehensive approach to domestic violence.
